# Cow Behavioural Activities in Extensive Farms: Challenges of Adopting Automatic Monitoring Systems

**DOI:** 10.3390/s23083828

**Published:** 2023-04-08

**Authors:** Dominga Mancuso, Giulia Castagnolo, Simona M. C. Porto

**Affiliations:** 1 Department of Agriculture, Food and Environment (Di3A), Building and Land Engineering Section, University of Catania, Via S. Sofia 100, 95123 Catania, Italy; dominga.mancuso@phd.unict.it (D.M.); simona.porto@unict.it (S.M.C.P.); 2 Department of Electrical, Electronic and Computer Engineering (DIEEI), University of Catania, Viale A. Doria 6, 95125 Catania, Italy

**Keywords:** precision livestock farming, Internet of Things, extensive farms

## Abstract

Animal welfare is becoming an increasingly important requirement in the livestock sector to improve, and therefore raise, the quality and healthiness of food production. By monitoring the behaviour of the animals, such as feeding, rumination, walking, and lying, it is possible to understand their physical and psychological status. Precision Livestock Farming (PLF) tools offer a good solution to assist the farmer in managing the herd, overcoming the limits of human control, and to react early in the case of animal health issues. The purpose of this review is to highlight a key concern that occurs in the design and validation of IoT-based systems created for monitoring grazing cows in extensive agricultural systems, since they have many more, and more complicated, problems than indoor farms. In this context, the most common concerns are related to the battery life of the devices, the sampling frequency to be used for data collection, the need for adequate service connection coverage and transmission range, the computational site, and the performance of the algorithm embedded in IoT-systems in terms of computational cost.

## 1. Introduction

“In recent years, animal welfare has become a central objective at a global level” (OIE Global Conference on Animal Welfare, 2004). Beyond the need for a common definition of animal welfare, the international scientific community has engaged in research aimed at improving the welfare of animals reared at various stages of production, from breeding to transport and slaughter, as well as developing and validating automatic systems for assessing animal welfare during the breeding process. In this regard, ICT-based monitoring systems have been created recently for the assessment of animal welfare at the farm level. These systems vary from one another in their specific task: to certify the level of well-being; evaluate the various housing systems; diagnose welfare problems on individual farms; and serve as a support tool for the breeder to find, prevent, or solve problems related to herd welfare. Consumers are also interested in achieving animal welfare because they value a proactive approach to managing animal health and welfare [1,2,3]. Recent research investigations have demonstrated that enabling dairy cows to express their natural behaviour in a natural setting is essential in the eyes of the public. Understanding how dairy cattle social behaviour relates to animal health, productivity, and welfare, as well as knowing farm managers’ and consumers’ perspectives on dairy cattle welfare and behaviour, allow for the development of management-relevant indicators for farmers’ decision-support systems, as well as the targeting of communication about “natural” animal behaviour to downstream chain actors, including consumers.

Animal behaviour is a clear sign of an animal’s physiological and physical state: cows’ major activities include feeding, rumination, lying, and walking, and their daily monitoring is crucial for farmers to evaluate cow welfare conditions. Operators can examine cows’ behavioural activities directly by visual examination, but it is a time-consuming and labour-intensive operation [4], especially on extensive farms. In the scientific field of precision livestock farming (PLF), ICT-based solutions are being developed and validated to increase the efficiency of livestock monitoring and management [5]. Such modern ICT-based solutions are becoming more and more efficient, ensuring the acquisition of a large amount of data that, when controlled by optimized algorithms, may be of tremendous assistance to farmers in monitoring the herd in an efficient and lucrative manner.

The fourth industrial revolution had a significant impact on industries and economic rules, as it allowed, through new technologies, the interconnection between machines, devices and people and laid the foundations for intelligent automation. This has led to a lesser presence of human help in the execution of repetitive actions, delegating these tasks to intelligent machines. Some of the pillars of the fourth industrial revolution are the Internet of Things (IoT), big data and analytics, autonomous robots, and cloud computing [6].

In recent years, solutions that integrate IoT systems with artificial intelligence techniques have been increasingly present. Advanced AI techniques have proved to be an efficient tool in the analysis of the large amount of data acquired from sensors producing new knowledge that cannot be obtained through traditional techniques [7,8].

The IoT is quickly evolving in the field of PLF. IoT-based systems connect computing devices, mechanical and digital equipment, items, animals, or humans to a network and transfer data without requiring human-to-human or human-to-computer contact [9].

**Figure 1:**
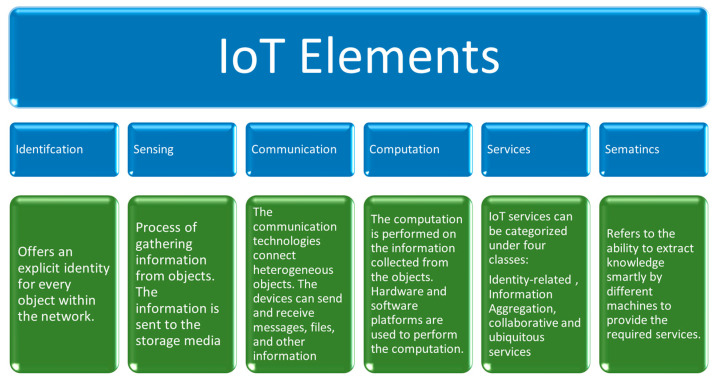
Main elements of an IoT system [9].

The main elements of an IoT-based system are object identification, sensing, communication, computation, service, and semantics, as described in Figure 1.

Farmers’ animal management practices have clear limits in the context of cow breeding. Most existing solutions are time-consuming, labour-intensive, and hence costly. Many livestock producers rely on stockperson observations to discover health and welfare concerns, although many commercial facilities have high stockperson-to-animal ratios.

The three main hurdles to efficiently monitoring cow welfare are cost, validity, and timeliness of insights [10].

The use of IoT-based sensors enables the early diagnosis of cow sickness, allowing farmers to intervene earlier and optimize antibiotic administration, milk supply, and veterinary care costs [11].

As a result, the use of wearable sensors is becoming a critical tool for monitoring the health and well-being of cows in housed systems. In most situations, such technology consists of (i) a device (sensor) that measures certain parameters; and (ii) software that processes the sensor’s data, generating information, warnings, and suggestions for the breeder [5]. Monitoring changes in cow behaviour with IoT-based wearable sensors provides unique insights into the study of an animal’s condition and well-being. Such changes may be caused by health and welfare issues, as well as dangers and changes in their environment [12]. Farmers can now monitor vital indicators, including blood pressure, heart rate, and hormonal levels; animal behaviours, including feeding, standing, rumination, and walking [13]; abnormal food and water consumption behaviours, e.g., limited feed ingestion influenced by unappetizing pasture quality, ruminal inactivity, and excessive water consumption due to increased walking activity [14]; and other parameters, such as geolocation information that can be recorded and further analysed [15]. Obviously, by knowing the time spent by animals on each behaviour, it is possible to carry out assessments of their state of health.

Only a few research projects have focused on extensive livestock systems, although IoT-based solutions for monitoring cow behaviour and well-being have been created exclusively in intensive housing systems. In this latter situation, the grazing animal monitoring faces several issues connected to the expansion of the grazing area and the animals’ ability to show their natural behaviour. Furthermore, because there is less human oversight, it is difficult to monitor and analyse the reasons for any unusual behaviour.

**Figure 2:**
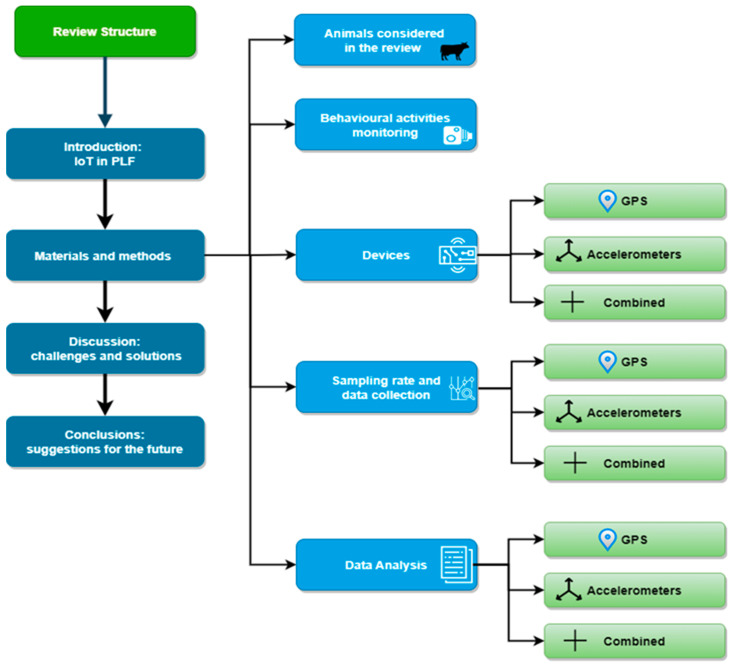
Review structure.

The aim of this review, structured as reported in Figure 2, is to highlight the main challenges that arise in the design and validation of IoT-based systems developed for monitoring grazing cows in extensive agricultural systems, because the issues to be faced are many and more complex than those encountered with animals housed in intensive systems.

A wide range of literature reviews revealed the most common concerns, including device battery life, sampling frequency for data collection, the need for adequate service connection coverage and transmission range, and the performance of the algorithm embedded in IoT-systems in terms of detection accuracy and computational cost [16,17,18].

## 2. Materials and Methods

The examination of published articles focused on the monitoring of cattle behaviour was carried out from January 2022 until July 2022, by using electronic repositories such as Web of Science, Scopus, Science Direct and Google Scholar. The articles analysed cover a timespan of about 16 years, from 2006 to 2022. The keywords used were “cow behaviour monitoring”, “animal welfare”, “monitoring cows in extensive farms”, “cow sensors”, “cow accelerometers”, “cow Global Positioning System”, “PLF and Internet of Things”. Only articles focused on research studies related to the monitoring of ruminants were selected, and among them, a further selection was made by considering only those carried out on the monitoring and analysis of cattle behaviour. The focus was placed on articles dealing with cow monitoring systems in extensive farms; however, some interesting systems tested in indoor farms were also considered as they could also be used in extensive farms.

### 2.1. Animals Considered in the Review

PLF research begins with the assumption that, while direct visual observation of animals by ethologists is critical for evaluating animal healthiness, it also produces findings that take, on average a long time, with substantial economic effort [19]. The large number of work hours necessary for visual monitoring means substantial expenses; hence, the adoption of ICT systems based on wearable sensors is becoming increasingly popular. However, the sensors in the devices should be constructed to avoid injuring animals, since this might impact their behaviour [20].

PLF has a wide range of applications, from continuous animal monitoring to environmental issue surveillance, whether for a single subject or an entire herd [21]. PLF may be used in many forms of farming, such as monitoring the scratching behaviour of laying hens within the cage, analysing the frequencies of vocalizations made during their productive life, quantifying animal weight, and predicting slaughter weight. PLF may be utilized in cow breeding to monitor the movements of the animals within the farrowing boxes in the hours before the calf’s delivery, as well as to diagnose laminitis events by analysing the animals’ locomotor profiles [22]. The most recent PLF applications in the cattle field involve the use of software for herd management and data collected by autonomous wearable sensors. Herd management software allows for real-time monitoring of cows on the farm, allowing for the identification of specific behaviours (such as feeding, drinking, lying, and rumination) with the goal of assessing the presence of heat, the relationship between food ingestion and milk production, and the level of well-being of the bred animals.

In the selected articles focused on the monitoring of cattle behaviours, approximately 80% used Holsteins, 10% unspecified crosses, Japanese Black Beef Cattle, and Angus, and the remaining 10% unspecified breeds. The average number of animals utilized in the study was around 30; however, this varied between studies.

### 2.2. Behavioural Activities Monitoring

As mentioned in the previous paragraphs, monitoring animal behaviour is critical for measuring animal welfare as well as successful herd management, particularly in extensive grazing systems. In this regard, ongoing automated behaviour analysis is a critical task since farmer-to-animal interaction is likely to be less frequent than in indoor breeding systems. As a result, today’s extensive farms are broader than in the past. It is not always easy to monitor animal behaviour by direct visual inspection by farmers.

In the articles found in the literature, several behavioural activities of cows were monitored:

- locomotion: helpful for identifying cow fertility, which is characterized by an increase in walking activity [23,24];
- feeding: a good indication of cow well-being since unwell cows eat less [25];
- rumination: a crucial phase of the digestive process [26] that is distinguished by a continual rhythmic chewing activity. Chewing action contributes to rumen pH remaining at a level optimal for microbial activity [26];
- lying: cows can be monitored for limb abnormalities by lying for lengthy periods of time in the absence of movement [27]

### 2.3. Devices

Sensors have played an important role in improving agricultural conditions and, more broadly, farm management since the introduction of PLF. Furthermore, with the advancement of increasingly effective IoT-based technologies, it has become feasible to employ sensor networks even in hostile settings such as barns, which are characterized by dust, a lack of energy, and a lack of an internet network. The literature review showed that there are various ICT-based monitoring systems developed for cows kept in indoor systems, but relatively only limited applications in extensive grazing farms. This is most likely due to the difficulties of employing ICT-based monitoring systems in rural locations where telecommunication network coverage is typically poor. Furthermore, the use of wearable sensors powered by batteries may result in management and maintenance expenses for farmers if the ICT-based systems are not optimized for energy savings [23]. 

Wearable sensors can collect a significant volume of data, as well as evaluate the raw data and inform farmers if the cattle’s behaviour is odd within a certain range [11].

In animal husbandry, several biometric and biological available sensors are often used, and they can be classified as non-invasive or invasive, as reported in Neethirajan et al. [10].

Invasive sensors are generally ingested or implanted in the animal’s body for tracking physiological measurements such as internal temperature. Non-invasive sensors are generally applied to an animal’s body by using collars or other attachment system to monitor livestock behaviour; moreover, non-invasive sensors are often installed in the breeding environment to monitor environmental parameters such as air temperature, relative humidity, and ventilation.

Since invasive sensors can directly measure animal health factors, they generate more accurate data than non-invasive sensors. However, non-invasive sensors are the most used macro-category because they are easily worn, have a lower cost, can be reused, and, most importantly, they cause less stress to animals than invasive ones.

Table 1 contains the major non-invasive sensors utilized in PLF applications, as well as the animal aspects monitored. Cameras and accelerometers are the most frequent non-invasive sensors used to monitor cow behaviour in indoor environments. Video-recording systems are low-cost solutions to observe the behavioural activities of several animals at the same time with a small number of cameras. An issue to consider when employing such non-invasive systems is animal identification, which is challenging and not always possible, even with the most advanced computer vision-based methods. Obviously, in extensive farms, it is not possible to install efficient video surveillance systems, due to both the extent of the grazing areas and unavailability of a constant and reliable energy source.

However, in recent years, with the introduction of increasingly efficient unmanned aerial vehicles (UAVs) equipped with cameras, some animal monitoring systems in extensive pastures based on the use of UAVs have been proposed [28,29]. The UAV-based systems just mentioned still needs improvement and development, as the limitation, to date, is in the very short battery life.

The non-invasive sensors most used in the field of cow monitoring in extensive farms are GPS (Global Positioning System) and accelerometers. Examples of GPS-based and accelerometer-based monitoring systems are reported in Table 2.

**Figure 3:**
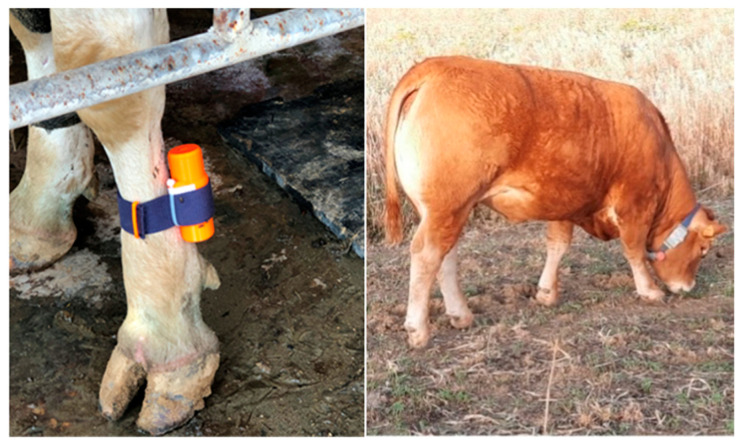
Example of pedometer and collar worn by cows [30,31].

Accelerometer-based systems are extremely flexible and inexpensive. They can be installed in an animal’s leg or neck to monitor behavioural activity (Figure 3). Pedometers are often attached to barn animals in the distal portion of the left hind limb and monitor the number of steps made by the cow; collars are attached to the neck and monitor head movements.

GPS sensors are used to locate grazing animals in extensive breeding systems; as in the case of accelerometers, they, too, can be worn by the animal by using collars. GPS is beneficial in those situations when the territorial extent of the grazing grounds does not allow for frequent and exact management of the herd.

For example, GPS devices can be used to decrease the likelihood of theft, defend against animal trespassing, and rescue injured animals when they are no longer able to move. The key challenge limiting GPS application to experimental domains is the short battery life of devices equipped with GPS sensors.

#### 2.3.1. GPS-Based Monitoring Systems

In several places in the world, GPS devices have been used to prevent cattle theft. In one study, a GPS collar was linked to the Global Mobile Communication System (GSM), and animals were followed by using a software application that informed the farmer when an animal left its grazing area, which was represented by a virtual fence [18].

Porto et al. [31] proposed a technique for tracking cows of a cow–calf line by using GPS, which allows for the collecting of data obtained at 20 min intervals. In particular, the gadget employed in the study includes the following characteristics: the GPS system and receiver are omnidirectional, with an integrated antenna, a temperature sensor, and a high-capacity Li-SOCL2 battery. The purpose was to identify the animals’ positions and determine the preferred regions of agricultural land where the animals spend the most time. Cow monitoring and location are critical pieces of information for studying the environmental implications of grazing cows as well as improving regular farm management. It is also feasible to identify in real time the cow’s oestrus period, which is marked by an increase in walking activity, or to tackle the problem of animal theft using GPS-based devices.

Hassan-Vàsquez et al. [32] studied the environmental effect of livestock production; the goal of this study was to assess the capacity of commercial GPS collar data, combined with farm features and meteorological conditions, to define the distribution of cow dung in paddocks. Seven animals were tracked by using commercial GPS tracking collars that included a GPS unit, a lithium battery pack, and a Sigfox communications module. These collars transmitted animals’ positions to the server in near real-time. If Sigfox coverage was available, GPS devices were set to obtain a location fix for each animal every 30 min.

Miliward et al. [33] studied cattle distribution over the landscape by using a GPS tracking-based system. The final aim was to assess the suitability of the guidelines proposed by Holecheck et al. [34], developed in order to help farmers to manage the stocking rate.

The communications network utilized is a crucial issue in this type of application, as some portions of the world, particularly rural areas, are currently underserved by efficient and dependable telecommunication networks [31].

#### 2.3.2. Accelerometer-Based Monitoring Systems

Several automatic monitoring systems based on the use of accelerometers have been created in state-of-the-art PLF applications, some of which are briefly detailed in Table 2.

Simanungkalit et al. [35] investigated the capacity of an ear tag accelerometer to detect licking behaviour at a block supplement in grazing cattle and validated the length of individual licking state predicted by an accelerometer and a system radio frequency identification system (RFID). Four Angus steers were fitted with an ear tag carrying a three-axis accelerometer.

In Riaboff et al. [24], the aim was the development of a framework for the prediction of cows’ behaviours, such as grazing, walking, rumination while lying, rumination while standing and resting while lying, by using 3-axis accelerometer data. The experimental trial was carried out on 4 different farms, and the considered number of cows was 86.

Benaissa et al. [36] provide an example of an animal monitoring system in which a new simple decision tree algorithm was developed for real-time classification of feeding and ruminating behaviours of dairy cows. In detail, the data used as model input were collected by using a neck-mounted accelerometer. Each cow was fitted with two devices: a RumiWatch (Agroscope, Switzerland) halter and an accelerometer. A RumiWatch halter is intended to be a measurement instrument for automated ruminant health monitoring.

A study of Y. Peng et al. [25] developed a recurrent neural network (RNN) model to detect and recognize calving-related behaviours by using inertial measurement unit sensors (IMU), i.e., a 3-axis accelerometer, a 3-axis gyroscope, a 3-axis magnetometer, and a wireless Bluetooth connection. The IMU data were collected from three expectant Japanese Black Beef Cattle that were placed in two barns in pairs and fitted with the same type of IMU sensor [37] attached to the collars. The RNN classified behaviour patterns, such as feeding, ruminating (lying), ruminating (standing), lying, and standing. Moreover, the RNN classified lying and standing behavioural activities during the last 24 h before calving, since they are generally modified when the calving is approaching. The monitoring of the calving is very important in extensive systems, where there is the highest mortality rate of calves due to a lack of immediate assistance during difficult calving.

Smith et al. [38] utilized behaviour monitoring collars with a 20-channel GPS, a 915 MHz microprocessor and transmitter, a 4 GB micro-SD card for data storage, and a Honeywell compass module HMC6343 with a 3-axis MEMS accelerometer and a 3-axis magneto resistive sensor installed on dairy cows (magnetometer). The inertial measuring unit was the compass module of the behaviour monitoring collars (IMU). The acceleration was measured in a three-axis inertial and gravitational system, with the *x*-axis sensing forward–reverse, the *y*-axis sensing left–right, and the *z*-axis sensing up–down. The device proposed by Smith et al. [38] was used to perform behaviour classification by using only the accelerometer data.

#### 2.3.3. GPS and Accelerometer Combined Systems

The use of GPS sensors is not sufficient to understand the behavioural activity of grazing animals; therefore, over the years, other systems have been proposed by combining mainly GPS sensors and accelerometers. The integration of motion sensors in GPS devices can provide the best method for evaluating some behavioural activities, such as feeding, walking, and lying, in relation to the grazing conditions.

As reported by Bailey et al. [39], the combination of GPS monitoring and accelerometers appears to be useful for recognizing changes in animal behaviour connected with livestock illnesses and other welfare problems. Such integrated systems might help farmers in making agricultural management decisions, such as food additions.

Most studies to date have focused on the use of GPS collars with accelerometers in small pastures and during short time periods; bigger pastures and longer time periods are needed to evaluate this technology.

Brennan et al. [15] wanted to determine whether a low-cost, non-commercial, lab-built GPS collar paired with a high frequency 3-axis accelerometer could predict regular cattle behaviour.

Riaboff et al. [40] investigated the relationship between the behaviour of grazing cows and pasture characteristics by using a combination of accelerometer and GPS data. They were acquired through an RF-Track 3D accelerometer and GPS sensor attached to the collar on the cow.

Various machine learning algorithms were used by Dutta et al. [41] to categorize cattle behaviour patterns collected by using collar systems with a 3-axis accelerometer, magnetometer, and GPS fitted to individual dairy cows to infer their physical behaviours. This study demonstrated that by using supervised machine learning techniques, cow behaviours can be categorized with great accuracy.

### 2.4. Sampling Rate and Data Collection

#### 2.4.1. GPS-Based Monitoring Systems

In GPS-based monitoring systems, the time acquisition interval affects the precision of the distance travelled by cows, the battery life, and how quickly farmers can respond to theft and trespassing [42]. If the device sends the positions at very long intervals, the risk is that any data loss could negatively affect the monitoring, so as to make the device inefficient. The sampling intervals normally used for GPS-based devices are between 1 and 60 min.

The system developed by Porto et al. [31] guaranteed long-term monitoring of the animals by allowing a collection of waypoints, such as latitude and longitude, of the cows selected in the study, the date and time of the survey, and the distance travelled by each animal. The time interval of data acquisition was set at 20 min to both ensure a long battery life and make possible further analyses carried out in a Geographical Information Systems (GIS) environment; i.e., the application of Kernel Density Estimation (KDE) algorithms. After receiving position information, the device sent it to a cloud server by using the Sigfox telecommunication network.

In Tangorra et al. [43], a GPS/GSM collar prototype was worn, using suggested commercial hardware and customized software, in order to follow animals’ movements beyond their grazing area and to alert when animals trespassed outside virtual perimeters. A standard customizable embedded firmware layer provided support for the hardware layer components. In fact, by using the created program, it was feasible to set the GPS acquisition interval from 1 s to 1 h.

Maroto-Molina et al. [44] developed a low-cost IoT-based system to monitor the location of each head of the herd. The system was based on GPS collars connected to a Sigfox network and low-cost Bluetooth tags. To preserve the battery life, the collars were configured to send data at a temporal resolution of 30 min. This time interval was reduced in Millward et al. [33], where GPS-sensors were set to acquire and send animal locations at 5, 10, or 15 min intervals.

#### 2.4.2. Accelerometer-Based Monitoring Systems

The sampling rate of the accelerometers in devices that monitor animal behavioural activities affects not only battery life but also the ability to correctly determine some behaviours. High sampling rates make it possible to acquire a lot of information, and therefore have more samples; however, they negatively affect battery life. On the other hand, low sampling rates help to preserve battery life but do not always allow the acquisition of good-quality data for classification purposes, as proved in Benaissa et al. [36].

In the studies reported in the literature, the accelerometer sampling rate ranges between 1 Hz [26] and 100 Hz [45]. Most of the works focused on a sampling frequency value between 10 Hz and 25 Hz.

A sampling frequency of 20 Hz is frequent, as demonstrated by Y. Peng et al. [25], who fitted each IMU sensor with a wireless Bluetooth connection to a computer. The IMU sensor was set to collect the 9-axis data points and send them instantaneously at 20 Hz. The IMU sensor’s battery life was about one week.

In Riaboff et al. [24], a sampling frequency of 59.5 Hz was used, and the total period of observation was about 3–4 days.

In Simanungkalit et al. [35], the battery life was a little bit longer. Four 3-dimensional accelerometers with a sampling rate of 25 Hz were used in their study. Each accelerometer was embedded into an ear tag. The ear tags were removed at the end of the trial, and the data were downloaded by using the proprietary software. The expected battery life for this setting was around 28 days.

In Smith et al. [38], the collars were set to collect accelerometer data at 10 Hz. The accelerometer data were saved on an onboard 4 GB micro-SD card and then downloaded when the trial ended. The effective battery life was approximately 14 days.

Recently, studies operating at frequencies of 4 Hz [46,47] have been reported. Raw data were acquired by collars embedded with a three-axis accelerometer and then sent to a cloud service by using a GPRS telecommunications network, which is known to be very energy consuming. The battery life of the proposed device was about 7 days; however, repetitions of the trials after the recharge of the devices made it possible to collect data for the classification of several behavioural activities, such as walking, feeding while walking, feeding in a standing position, rumination, and rumination in a lying position.

#### 2.4.3. GPS and Accelerometer Combined Systems

Some research studies described the development of combined systems, i.e., devices embedded with accelerometers and GPS sensors.

González et al. [48] used collars for monitoring cow location and behavioural activities by using GPS data acquired at 4 Hz (i.e., 345,000 data points/day) and accelerometer data at 10 Hz (i.e., 862,500 data points/day). Data were saved in a memory storage card installed in the devices and downloaded at the end of the trial. The battery life of the device was around 12–14 days.

Dutta et al. [41] employed location and behavioural tracking collars in their study (GPS, 3-axis accelerometer, 3-axis magneto resistive sensor, and 4 GB micro-SD card for data storage). GPS data were obtained at 4 Hz, while accelerometer data were recorded at 10 Hz. Data on grazing behaviour were collected by using the WhatISee digital application. After the experiment, the data were saved on an SD card and downloaded. The battery life was around 12 days.

Brennan et al. [15] fitted tracking collars with a GPS data logging device and a high-frequency accelerometer. The GPS logger was set to gather a fix (latitude/longitude) every one minute. The accelerometers were programmed to capture data at a rate of 12 Hz. The accelerometer data were saved on an onboard 8 GB micro-SD card and then downloaded when the trial ended. The battery life was around 50 days by using two independent batteries.

In Riaboff et al. [40] fitted 26 cows with a 3D accelerometer and a GPS sensor mounted to a collar. The accelerometer data were paired with GPS data to predict the behaviours every 10 s. The data in this study were saved on a secure digital card and had to be manually extracted. The sampling frequency of the accelerometer was 59.5 Hz and that of the GPS was 1 Hz. The system’s battery life was barely 5 days.

In Cabezas et al. [49], the animals wore a low-cost commercial device equipped with 3D accelerometers and GPS sensors. The accelerometer sampling frequency used was 10 Hz and the GPS acquisition interval was set to 5 min. The battery life was approximately 2–3 months.

In the research studies reported above, data were mainly stored in memory cards. It is obvious that automatic data transfer through a telecommunication network would be more convenient for a long-term experiment and, above all, for practical use by farmers.

**Table 2:** Main analysed IoT systems designed to perform animal monitoring.

Devices	No. Monitored Cows	Sampling Rate/Time Interval	Data Collection and Storage	Aim	Monitoring Period	References
GPS	10	20 min	Sigfox/On Cloud	Cow position tracking	45 days	[31]
GPS	6	10 min	Sigfox/On Cloud	Cow position tracking	45 days	[50]
GPS	7	30 min	Sigfox/On Cloud	Cow position tracking	5 months	[32]
GPS	180	5/10/15 min	On Device	Cow position tracking	From 1 to 4 months	[33]
GPS	5	-	GSM	Virtual fencing	5 months	[43]
GPS	50	30 min	Sigfox and Bluetooth/On Cloud	Cow position tracking	3 months	[44]
Accelerometer	12	25 Hz	On Device	Detect licking behaviour	28 days	[35]
Accelerometer	10	10 Hz	On Device	Detect feeding and rumination	5 days	[36]
IMU sensor	3	20 Hz	Bluetooth/External PC	Classify feeding, rumination, lying, and standing	7 days	[25]
Accelerometer	4	4 Hz	GSM/On Cloud	Classify feeding, rumination, walking, and lying	7 days	[46,47]
Accelerometer	86	59.5 Hz	On Device	Classify six different behaviours	3–4 days	[24]
Accelerometer	24	Acc. 10 Hz	On Device	Monitor cows’ behavioural activities	14 days	[38]
GPS + Accelerometer	5	Acc. 12 Hz/GPS 1 min	On Device	Predict animal behaviour and animal tracking	3 months	[15]
GPS + Accelerometer	26	Acc. 59.5 Hz/GPS 1 Hz	On Device	Understand relation between behaviour and pasture characteristics	5 days	[40]
GPS + Accelerometer	24	Acc. 10 Hz/GPS 4 Hz	On Device	Classify animals’ behaviours	12 days	[41]
GPS + Accelerometer	14	Acc. 10 Hz/GPS 4 Hz	On Device	Monitor cows’ location and behavioural activities	12–14 days	[48]
GPS + Accelerometer	30	Acc. 10 Hz/GPS 5 min	On Cloud	Monitor tracking movement and tracking location	2–3 months	[49]

### 2.5. Data Analysis

#### 2.5.1. GPS-Based Monitoring Systems

The data collected by GPS sensors are mainly analysed by using statistical and geo-spatial tools, such as GIS tools.

In Porto et al. [31], all information was transmitted to a custom AppWeb operating on mobile devices or a personal computer. The data were then imported and processed by using statistical and geospatial analysis. In detail, to better understand the interaction between livestock activities and the environment, spatial analyses were carried out by using the QGIS software, which allowed data processing and visualization at the territorial level. Analyses on the location of each animal equipped with the created devices were performed by using the KDE tool. In particular, the KDE analyses allowed the computation of the home range of the species and offered an estimate of the territorial areas mostly occupied by the animals.

Recently, clustering methods, which are part of the unsupervised machine learning (UML) category, have been used to process the data acquired through GPS sensors.

In Xu et al. [51], unsupervised machine learning algorithms were used to analyse location data in order to understand the social structure of a small group of cattle and an individual’s social behaviour. Based on logical and physical distance, k-means clustering was employed. The leader animals and their influence on an individual in a cattle herd were discovered by comparing the clustering results based on logical distance and physical distance, which provides significant information for understanding animal herd behaviour.

#### 2.5.2. Accelerometer-Based Systems

Through the sensors described in previous Section 2.3, the data required for studying animal behavioural activities were acquired during the experimental trials and then processed. The main data elaboration phases have been identified and reported here, as follows:

- Pre-processing that usually included:
  ○ Filtering: to remove noise or minor behaviours.
  ○ Data augmentation: techniques used to increase, in an artificial manner, the amount of data by adding slightly modified copies of the original data.
  ○ Grouping samples in windows: to extract more significant information from groups of samples instead of a single sample.
  ○ Feature selection and computation: to select and compute the subset of relevant features which were used in model building.
  ○ Dataset splitting in subset: to determine which portion of the dataset to train and on which portion to test the performance of the developed model.
- Recognition: consists in the classification of the behaviours of the interested parties using the information acquired in the previous steps. The recognition process is performed by a specific model or method. In the state-of-the-art applications, different methods were used: identification of thresholds, statistical analyses, and, more recently, application of machine and deep learning techniques.

Over the years, various techniques have been used to process the acquired data and, thus, to be able to draw knowledge from them. As previously mentioned, most of the studies have been focused on the use of accelerometers; the data collected through the latter show acceleration values for each instant in time, so the data collected were time series. There are several methods for processing time series; however, the most used to process the data acquired through accelerometers in the PLF research field can be grouped into six main categories [52] (Table 3):

- Statistical Model (SM): SM provides a set of statistical assumptions about how sample data are generated. Typically, a statistical model is defined as a mathematical relationship between one or more random variables and other non-random variables.
- Manual Thresholding (MT): MT is widely used in the real world, since it has the advantage of requiring simple calculations, which is important for devices with low computational capabilities and energy-saving requirements. The thresholds were determined by descriptive statistics, such as medians, means, maximum, and minimum, carried out on the acquired dataset.
- Machine Learning (ML): ML is a subset of artificial intelligence (AI) that concerns the creation of systems that learn or improve their performance based on the data they use. There are three sub-categories of ML:
  ○ Supervised Machine Learning (SML): SML consists in using, as input to the model, labelled data; i.e., the label summarizes the nature of that data. Usually, tasks relating to classification are tackled by using supervised approaches.
  ○ Unsupervised Machine Learning (UML): UML consists in using input data that have no label; thus, an UML algorithm that inputs unlabelled data will reclassify and organize the inputs based on common features to try to make statistical predictions about future inputs. Clearly, it is applied to the extraction of information not yet known.
  ○ Supervised Ensemble Machine Learning (SEML): SEML is a collection of algorithms used to improve overall performance by combining the predictions from multiple models.
- Deep Learning (DL) is a type of machine learning based on artificial neural networks (ANN), in which multiple processing layers are used to extract progressively higher-level features from data. ANN are inspired by the way the human brain analyses information and learns. In recent years, DL algorithms have become very popular because they make it possible to reach good performance in classification problems. However, is not always possible to apply them in firmware embedded in IoT-based systems as they require large computational resources and large amounts of data to be processed. The most used ANN are reported here, as follows:
  ○ Multi-layer perceptron (MLP): a feedforward ANN that consists of an input layer of neurons, one or more hidden layers of neurons, and an output layer of neurons, where each layer is fully connected to the next layer.
  ○ Convolutional Neural Network (CNN): CNN, also known as ConvNet, is a type of feed forward neural network that excels at processing input with a grid-like architecture, such as images. To summarize the process, neurons in a CNN receive inputs, perform scalar products using weights learned throughout the training, and then apply a non-linearity function to the created result. The CNN’s distinctive aspect is the convolution layer, which divides the input into several little parts and then superimposes a filter called the kernel. As a result, each component can be used to extract features, or the main characteristics of the input data.
  ○ Recurrent Neural Network (RNN): This is a class of ANN, different from the previous, which provides the presence of cycles within the network; i.e., in certain layers, the output provided by the latter becomes the input for the same layer or for lower layers; i.e., there is feedback. The interconnection between layers allows the use of one of them as a state memory, and allows, by supplying a temporal sequence of values as input, the modelling of a dynamic temporal behaviour dependent on the information received at previous instants in time.

#### 2.5.3. GPS and Accelerometer Combined Systems

In the studies found in the literature, the analysis of data collected by devices that are equipped with GPS and accelerometer sensors was carried out by using mainly statistical models and machine learning methods, as previously reported for the GPS and accelerometer sections.

Gonzàlez L. et al. [48] developed an algorithm which classified data acquired from collars into five behavioural activities. The aim of the study was to obtain the proportion of the daily time that individual animals spent on each activity. They employed two different datasets in the experimental trial, where data from accelerometers and GPS were first aggregated by computing the mean and standard deviation (SD) over 10 s time intervals. The first dataset, which included a subset of data in which behavioural activities were identified based on visual observations, was used to determine differences between activities from sensor data values; to inspect frequency distributions (histograms) of data with different activities; to select variables suitable for decision trees; and to construct conceptual decision trees. The second dataset, containing all data related to unknown behavioural activities, was used to fit probability density functions in mixture models that determined threshold values to separate populations of data points.

The aim of Cabezas et al. [49] was to develop a general approach to recognizing numerous activities based on accelerometer and GPS sensor data. The accelerometer signals were collected, and the data from each axis were individually analysed to extract 108 temporal and frequency domain properties. A total of 238 activity patterns, corresponding to grazing, ruminating, laying, and steady standing, were captured on video and matched with raw accelerometer data. Accelerometer signal features were utilized to train a Random Forest (RF) algorithm for behavioural pattern categorization, and GPS position data were analysed by using an UML technique to discover abnormal activity patterns. The clustering method k-medois was adopted to analyse the GPS data; the authors chose k-medois over k-means because k-medois is more stable in the presence of outliers.

Dutta et al. [26] proposed the combination of a temperature sensor, a GPS module, and a 3-axis accelerometer. The datasets for all the animals were used on each day of the study, and all the datasets acquired were appropriately integrated and used without any filtering. By following the data collection phase, the most important attributes were selectively extracted for improving data interpretation. Each dataset included sensor values for temperature, walking speed, and acceleration along the X, Y, and Z axes. Extreme Gradient Boosting (XGBoost) and Random Forest classifiers were used to categorize behaviours like ‘standing’, ‘lying’, ‘standing and ruminating’, ‘lying and ruminating’, ‘walking’, and ‘walking and grazing’.

To predict behaviour, Brennan et al. [15] choose four classification algorithms: Random Forest (RF), Linear Discriminant Analysis (LDA), Quadratic Discriminate Analysis (QDA), and Support Vector Machines (SVM). The response variable was livestock behaviour, and the predictors were metrics derived from accelerometer and GPS devices. The datasets utilized to generate these models only comprised data by including observational, GPS, and accelerometer data. Algorithms were adopted to categorize behaviour as graze or non-graze. A validation set technique was used to verify the accuracy of each model by randomly partitioning each dataset into an 80% training, 20% test dataset.

In Riaboff et al. [40], the goal was to investigate the relationship between cows’ behaviours and the pasture characteristics. The study can be divided in two steps: in the first, the cows’ behaviours were classified, and in the second were computed the time-budgets expressed in each zone by each cow per day and behaviour. The behaviour was predicted among six different classes by using the Extreme Gradient Boosting (XGB) proposed in a previous study [24]. The behaviour classification considered samples of accelerometer data grouped into windows. Afterwards, the predicted behaviours in successive windows from the same cow were smoothed by using the Hidden Markov Model (HMM)-based Viterbi algorithm. The predicted behaviours were combined with the GPS data and the time budgets expressed in each zone were calculated. In order to investigate the relation between the time budgets and pasture characteristics, a Linear Mixed Model (LMM) was used.

## 3. Discussion

The PLF technologies applied to monitor the behavioural activities of cows have found some common problems with their application in extensive farms, which imply a more restrictive use than the indoor environment. Although awareness of the potential of these tools is still limited, the demands of farmers and researchers are increasing, and positive results are expected from the spread of PLF in grazing systems in terms of animal welfare and work optimization.

Several studies in the literature have adopted GPS [16,61,62] for the localization of cattle, demonstrating the common need to solve problems related to miniaturization of sensor technologies and the development of high energy density batteries [50].

As reported by Raizman et al. [63], to extend the battery life of the devices, in some research studies, the position of the animal was detected only once an hour (or a little more), with the result that in reducing the number of detections, it is impossible to achieve an efficient monitoring of the grazing animals. Another method to improve battery life is to apply a standby/sleep mode that deactivates the device when the sensors it is not be used, in addition, adapting the alarm rate to the activity performed by the animal [46].

**Figure 4:**
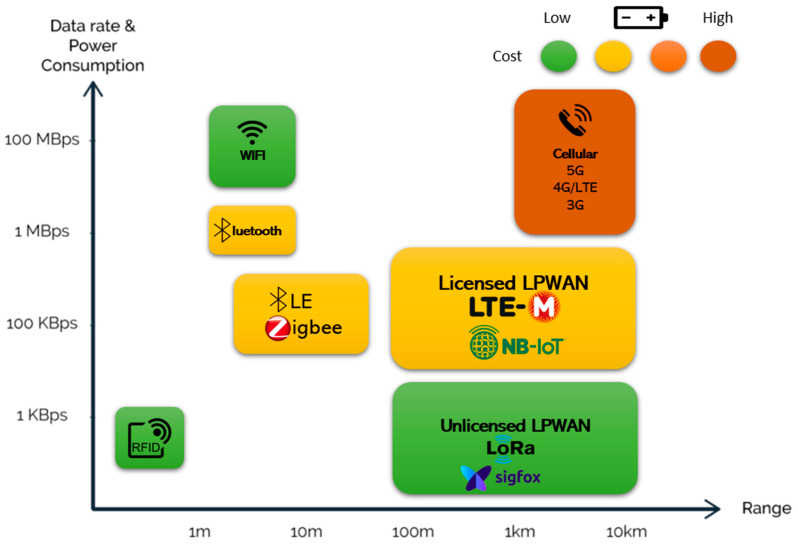
Communication techniques in IoT classified by data rate, power consumption, and range.

A critical problem with these types of applications is certainly the telecommunication network used, because there are vast areas of the world, in particular rural areas, where the coverage of an efficient and reliable telecommunication network is lacking [18]. In the specific case of mobile tracking systems for livestock, it is necessary to have a telecommunications network that is not associated with high power consumption as it may result in reduced battery life. To overcome the above problems, different Low-Power Wide-Area Networks (LPWAN) [64] have been proposed, which are types of long-range wireless telecommunication networks characterized by low power consumption and low bit rate (Figure 4). Sigfox and LoRa [65,66] are two of the most widely used LPWANs in IoT applications in PLF because they offer real-time and low-power monitoring of animals in extensive farms.

In Porto et al. [31] and Castagnolo et al. [50], the priorities were to study the battery life and the feasibility of a Sigfox-based tracking system in extensive farms. By using the Sigfox network, it was possible to have a battery life of about 4 months by using a 10 min time interval for data collection. Furthermore, the proposed system was tested in an area where the coverage of the telecommunications network was poor and by installing a repeater in the area, it was possible to continuously monitor the animals without incurring significant data loss.

The most recent, LoRaII, offers the potential to derive object position by triangulating the arrival time of packets via highly synchronized base stations. The position accuracy provided by LoRaII (10 m to 30 m) may be adequate for applications involving animals confined to a specific area (e.g., locating sick cows or farrowing), but may become inadequate when grazing areas are wider and not enclosed by fences.

An interesting application of devices embedding GPS sensors are virtual fences. These new technologies are aimed at confining grazing animals (e.g., cows, sheep) without physical fences. Virtual fences are based on animals’ associative learning by using audio signals and an electrical discharge delivered if the animal trespasses and does not change direction after the acoustic signal.

Since devices embedded with GPS sensors could also provide animal identification, each animal position could be tracked during the monitoring period and located within the grazing farm by software based on GIS [67].

The GPS data acquired through the various devices proposed in state-of-the-art applications are certainly important, because by knowing the position of the animal, it is possible to reduce the probability of theft or trespassing. However, by managing GPS data within GIS tools, it is possible to highlight crucial aspects, such as the environmental impact, the social aspects, and the forage ingested by animals.

Through the GPS data, if well known, it is possible to make evaluations about the habits of the animals, but it is not sufficient to give information about the behaviour of grazing animals.

The integration of motion sensors with GPS data can provide the best method for determining animal activity on extensive farms. These types of devices are an undeniable advantage for the farmer, who can be immediately alerted in case of abnormal behaviour [31]. Ongoing research has shown that IoT-based devices embedded with accelerometers can remotely monitor livestock behaviour and detect changes in activity associated with disease and calving. GPS tracking can also detect calving by monitoring the distance between a cow and the rest of the herd or identify when cattle gather in sensitive areas. Combinations of GPS and accelerometer data can be more accurate than either device used alone.

To date, studies that combine GPS data and accelerometers are a very low percentage [15,68], compared to works that use single types of data. In Cabezas et al. [49], as described above, an ML-based procedure to recognize multiple activities by using accelerometer and GPS data was proposed. The results obtained confirmed that the proposed methodology could be generalized to other behavioural models, but it is necessary to test the method by using further data samples, implementing the availability of labelled data, as well as improving battery life.

One of the biggest challenges in monitoring livestock behaviour by using sensors, but above all, by using combined systems, is the management of the large volume of generated data, as reported by Brennan et al. [15]. The collection of a large amount of data implies problems not only in management but also in processing and storage, as higher, and therefore more expensive, computational resources are required.

Nowadays, most studies have focused on the use of GPS collars embedded with accelerometers in small pastures over short periods of time; assessment of this technology on bigger pastures over longer periods of time is required. In Riaboff et al. [40], it was only tested for 5 days as the battery ran out. This period of observation, as evidenced by the authors of the work, is not enough to explore the relationship between cows and their environment, especially in herding, where cows graze continuously for a long period of time.

By analysing the studies present on the state of the art, it emerges that, when a new system and method of data processing is proposed, it is not always possible to make comparisons, since the software tools and the acquired data are not always shared. This does not allow a full understanding of the limits of the proposed studies, as well as a replicating of the results later.

**Figure 5:**
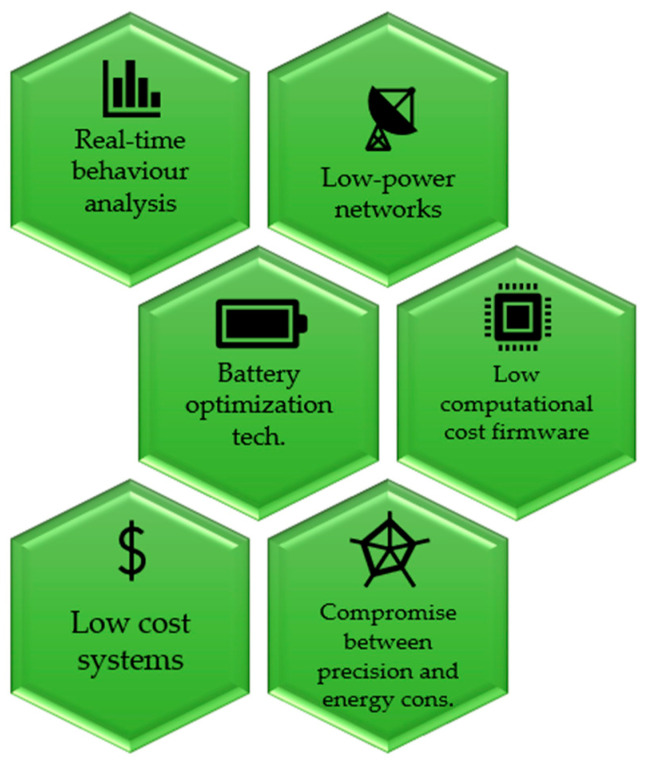
Future key aspects in the adoption of animal monitoring systems in extensive farms.

Ultimately, from the descriptions of the studies proposed above, it is clear that the aspects to be considered and investigated are how to improve both battery life and the reliability of the telecommunications network. Regardless of the existing technological limitations, the solutions to the first problem are to be found in the use of low-power telecommunications networks; the creation of highly optimized firmware aimed at energy saving; the energy optimization techniques of the devices; and a reduction of the sampling frequency in devices with the acquisition and use of high-efficiency batteries. Obviously, high acquisition frequencies involve greater energy consumption; however, they allow greater precision in behaviour detection, such as rumination or walking. Therefore, it is necessary to investigate a compromise between precision and energy consumption [24,53] (Figure 5).

As reported in Section 2.5, recently, ML and DL techniques have been proposed to perform cow activity detection, instead of traditional techniques based on statistical methods or threshold identification. ML and DL techniques, despite their higher computational cost, provide more generalizability and less human intervention in their design since discriminative features are learnt by the model itself. However, the need for more computational resources poses a challenge in terms of battery life. To solve this issue, the necessary computations are often carried on cloud platforms by using the telecommunications network to send the raw data acquired by the sensors.

This solution could be valuable to monitor cow behavioural activities in intensive indoor systems because the devices that transfer the data (gateway) acquired by the wearable sensors to the cloud could be fed by the electrical network. In extensive grazing systems, this solution is not always possible because data transfer to the cloud should be carried out by using GSM telecommunication networks that are highly energy consuming for the wearable devices. Though personal area networks (PAN) or local area networks (LAN) fed by electrical networks should be used to collect and then transfer data, they assure a short communication range, often not suitable when the grazing area is very extended. Finally, also, when LPWANs are used to transfer the data from sensors, their payload is generally small and does not allow transferring the big data required for ML and DL models.

Based on this observation, models working with pre-determined thresholds could be a solution for monitoring the behavioural activities of grazing cows in rural areas because threshold-based firmware with low computational costs could be implemented in the wearable devices, with the processed data transferred via an LPWAN. However, in order to have thresholds that can allow a good classification, it is necessary to have a lot of data.

As far as the reliability of the LPWAN telecommunications network is concerned, a possible solution to the coverage lacking in rural areas could be the installation of network repeaters, equipped with energy accumulators powered by energy from renewable sources such as wind or photovoltaic systems.

## 4. Conclusions

As previously mentioned, the combined systems, which have GPS and accelerometers, allow monitoring of the activities of grazing animals in a more complete way, compared to systems that use only one type of sensor. To date, it has emerged that in the state-of-the-art applications, various prototypes of GPS collars have been proposed which send the positions of the animals in real time, while in the case of accelerometers, the prototypes which send the data in real time are numerically smaller. In many studies, the data acquired with the accelerometer were downloaded at the end of the tests, as the objective of the studies was to propose methodologies for behaviour detection. Therefore, the data were not sent in real time. Sending raw data from real-time accelerometers requires a telecommunications network capable of transferring large amounts of data. If, instead, the computation is performed on-site, it is necessary to deal with the computational cost of the processing and the relative performance of the batteries.

In addition, as far as the combined systems are concerned, in this case, the devices that jointly process the data acquired by the accelerometer and GPS are very few. In this case, many of the acquired data are processed subsequently, and, moreover, not always jointly; i.e., two different processes are carried out based on the nature of the data.

In the future, it is hoped that researchers will focus on real-time behavioural analysis, using joint position and movement data, to respond to farmers’ needs. What has been said involves, in part, low-power networks, battery optimization techniques, and low-computational-cost firmware.

Animal monitoring systems, as reported in the previous sections, must therefore be able to work in rural areas, where the electricity grid and telecommunications network are not reliable. They must be simple to use and plug-and-play, and, very importantly, they must be low-cost. Costs have not been examined in this work as most of the analysed devices were prototypes; however, this aspect is crucial for farmers to adopt this type of system, especially when there are numerous animals to be monitored.

Greater collaboration among farmers, animal scientists, agronomists, engineers, and other experts would aid in the development of strong technology suitable for long-term use in the agricultural environment. The use of digital technology in livestock systems could provide useful support to investigate and completely understand the dynamics and impact of climate change on farm animal ecology, in addition to better manage grazing animal behaviour.

## Figures and Tables

**Table 1:** Non- invasive sensors in PLF applications.

Sensor/Device	Aspect of Animal	Disease/Used for
GPRS, GPS and transponders, accelerometers	Cattle’s position, inside the barn or outside the barn	Grazing, feeding, lying, behaviour and welfare monitoring
	Motion changes	Lameness, oestrus
Pressure	-	Feeding and drinking monitoring
Microphone, UHF sensors	Monitor sound levels in barns	Mooing, pain and welfare conditions, rumination, breathing disease
Temperature	Temperature monitor	Fever, ovarian cysts, pneumonia, retained placenta, mastitis
Thermal infrared camera, 2D cameras, 3D cameras	-	Behaviour monitoring, lameness, oestrus
Load sensor	Weight distribution	Lameness
Gas sensor	Breathe ketones, methane emission	Displaced abomasum, ketosis
Radio-frequency identification	Identification	Behaviour and welfare monitoring

**Table 3:** Methods used for the processing of data acquired through accelerometers to detect cow behaviour.

Technique	Sub-Type	Methods	References
Threshold methods			[46,53]
Statistical models	-	Logistic Regression (LR), Hidden Markov Models (HMM), Linear Mixed Models	[38,54]
Machine learning	Supervised	Linear Discriminant Analysis (LDA), Quadratic Discriminant Analysis (QDA), Support Vector Machine (SVM), k-Nearest Neighbour (k-NN), Naïve Bayes models (NB), Decision Trees (DT)	[55,56]
	Unsupervised	k-means	[57]
	Ensemble	Random Forest (RF), Extreme Gradient Boosting (XGB), Adaboost (ADA)	[24,58]
Deep learning		Multilayer Perceptions (MLP), Convolutional Neural Networks (CNN), Recurrent Neural Networks (RNN), Long short-term memory (LSTM)	[59,60]

## Data Availability

Not applicable.
